# Doping Tuned the Carrier Dynamics in Li-Doped Bi_2_Se_3_ Crystals Revealed by Femtosecond Transient Optical Spectroscopy

**DOI:** 10.3390/nano15131010

**Published:** 2025-06-30

**Authors:** Qiya Liu, Min Zhang, Xinsheng Yang, Tixian Zeng, Minghu Pan

**Affiliations:** 1 College of Optoelectronic Technology, Chengdu University of Information Technology, Chengdu 610225, China; liuqiya@cuit.edu.cn (Q.L.); zengtx@cuit.edu.cn (T.Z.); 2 Superconductivity and New Energy R&D Center, School of Physical Science and Technology, Southwest Jiaotong University, Chengdu 610031, China; 3 School of Physics and Astronomy, China West Normal University, Nanchong 637002, China; 4 Dazhou Industrial Technology Research Institute, Dazhou 635000, China; 5 School of Physics and Information, Shanxi Normal University, Xi’an 710119, China; minghupan@snnu.edu.cn

**Keywords:** topological insulators, femtosecond transient optical spectroscopy, carrier dynamics

## Abstract

Topological insulators (TIs) can be widely applied in the fields of ultrafast optical and spintronic devices owing to the existence of topologically protected gapless Dirac surface states. However, the study of ultrafast dynamics of carriers in TIs remains elusive. In this work, the carrier dynamics of Li-doped Bi_2−x_Se_3_ single crystals were investigated by femtosecond (fs) transient optical spectroscopy (ΔR/R(t) signals). The temperature dependence for the relaxation rates of the electron–electron interaction and electron–phonon coupling is consistent with the results of electrical transport, which indicates the carrier dynamics of TI is highly related with carrier concentrations. We find that the carrier type and concentration of Bi_2_Se_3_ can be tuned by Li doping, leading to a metal-insulation transition at low temperatures (T ≤ 55 K), indicating that electron–electron interactions are dominant at low temperature. For T > 55 K, electron–phonon coupling in the bulk carriers becomes the main electric transport mechanism.

## 1. Introduction

Bismuth selenide (Bi_2_Se_3_) is classified as a three-dimensional (3D) topological insulator (TI) and has attracted intense interest due to its sizable bulk band gap (~0.3 eV) and gapless metallic surface states [1,2,3], topologically protected by time reversal symmetry [4,5,6] and spin–orbit interactions [7,8]. The unique transport properties of the topological surface state (TSS) have broad potential applications in the fields of ultrafast optical and spintronic devices [9,10,11]. These tunable electronic high-speed devices can be designed based on the different mechanisms of electron energy relaxation in 3D and 2D states associated with the electron–phonon interaction and deformation potential/thermoelastic scattering, respectively [12,13,14,15,16]. Previously, the ultrafast dynamics of TIs has been reported. For example, the thickness dependence [16,17,18] and the temperature dependence [19,20,21,22] of ultrafast dynamics in TIs have been studied. Qi et al. [23] found that the ultrafast carrier and phonon dynamics of Bi_2_Se_3_ crystals are affected by the environment. As recently reported by Tu et al. [24] and Shi et al. [25], the ultrafast dynamics of Bi_2_Se_3_ are sensitive to impurities. These results indicate that the ultrafast dynamics of Bi_2_Se_3_ can be effectively tuned by multiple factors, suggesting that the topological insulator Bi_2_Se_3_ has broad potential application in the field of tunable optoelectronic devices. Understanding the ultrafast dynamics of Bi_2_Se_3_ is essential for future optoelectronic and spintronic applications.

In addition, bulk Bi_2_Se_3_ is actually poorly insulating due to the intrinsic Se vacancy. To achieve a bulk-insulating state in Bi_2_Se_3_, hole doping to compensate for the residual electrons is a viable strategy. Tremendous research interest in metal-doped TIs has been focused on p doping. For example, Mn doping can change the carrier type from n-type to p-type [26]. Cd doping can suppress the formation of Se vacancies and tune the carrier type and density in Bi_2_Se_3_, and achieve insulating behavior [27]. Doping of Nb, Sr, and Cu can induce superconductivity in Bi_2_Se_3_ [28]. The Fermi energy of Bi_2_Se_3_ can be tuned by 1% Mg doping to realize quantum topological transport [29]. However, with strong metallic properties, the light element of lithium (Li)-doped Bi_2_Se_3_ has not yet been reported. In previous studies, Li ions have been injected into layered materials and tuned the material properties [30,31,32]. Therefore, introducing Li into Bi_2_Se_3_ is feasible and may lead to change in the properties as well as the ultrafast dynamics of Bi_2_Se_3_.

In this work, Li_x_Bi_2−x_Se_3_ crystals were synthesized by the self-flux method. The crystal structure and surface morphology were characterized. The ultrafast dynamics of the bulk and surface states were investigated by both electrical transport measurements and femtosecond transient optical spectroscopy. The R-T curve and the data of Hall measurements show that the carriers in Bi_2_Se_3_ can be tuned by the Li impurities. Femtosecond (*fs*) transient optical spectroscopy (ΔR/R(t) signals) was measured and analyzed to obtain the ultrafast dynamics parameters. The temperature dependence of the relaxation rate of the electron–electron interaction and electron–phonon coupling is found to be consistent with the results of electrical transport. The results indicate that electron–electron interaction is dominated by surface state carriers at low temperatures (T ≤ 55 K), and electron–phonon coupling is dominated by bulk carriers at T > 55 K.

## 2. Experimental Section

Li_x_Bi_2−x_Se_3_ (x = 0, 0.02, 0.05 and 0.08) crystals were synthesized by melting high-purity (99.999%) bismuth, selenium powders, and Li particles (Aladdin, Shanghai, China) in their molar ratio at 850° C in a high-vacuum quartz tube. The evacuated quartz tubes were slowly (3.8° C/h) cooled to 620° C. Finally, the samples were quenched in cold water. The test samples were exfoliated flakes from the Li*_x_*Bi_2−*x*_Se_3_ block, the size ws~3.0 mm × 1.5 mm, and the thickness was about 650 nm (see the Supplementary Materials part S1).

The fresh surfaces for all samples were tested by X-ray diffraction (XRD, X’Pert Panalytical, Almelo, The Netherlands) with Cu Kα emission (λ = 1.5418Å). at room temperature. The microstructure was examined with field emission scanning electron microscopy (FESEM, JSM−7001F, Japan Electron Optics Laboratory Co., Ltd.). Electrical transport properties were examined by a Physical Property Measurement System (PPMS, Quantum Design, USA) at temperatures ranging from 5 K to 300 K using the standard four-probe method.

Using femtosecond (*fs*) pump-femtosecond (*fs*) probe spectroscopy (Coherent, USA), the Δ*R*/*R*_0_ (t) measurements were performed using a commercial mode-locked Ti: sapphire laser system (~35 *fs* pulse duration, 800 nm center wavelength, and 80 MHz repetition rate). Both pump and probe laser beams were focused onto the sample with a spot diameter of ~30 mm^2^. The pump and probe beam fluence was 10.0 J/cm^2^ and 1.00 J/cm^2^, respectively.

## 3. Results and Discussion

**Figure 1:**
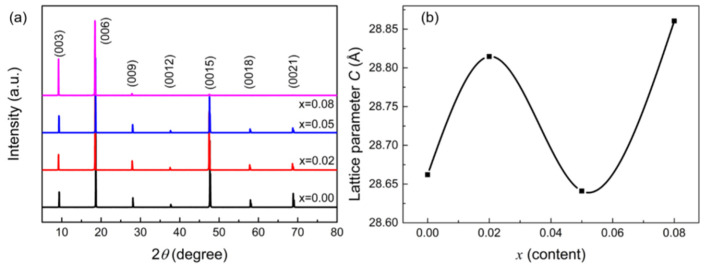
(**a**) The crystal structure of Li_x_Bi_2−x_Se_3._ (**b**) The lattice parameter *c* of Li_x_Bi_2−x_Se_3_.

Figure 1a shows the XRD patterns for the Li_x_Bi_2−x_Se_3_ crystals. All the diffraction peaks are attributed to the (0 0 *l*) orientation of Bi_2_Se_3_ without an obvious impurity phase, which indicates that the samples have a pure *c*-axis orientation. The lattice constants of the *c*-axis were calculated, as shown in Figure 1b. With Li contents ranging from 0 to 0.08, the parameter *c* increases from 28.66 Å, 28.82 Å, 28.63 Å and 28.86 Å. The results indicate that Li atoms are mixed into the Bi_2_Se_3_ crystal.

Simultaneously, we also provided the ICP-OES data in the Supplementary Materials, and the results of ICP-OES indicated that the content of Li was consistent with the stoichiometric ratio in the Li*_x_*Bi_2−*x*_Se_3_ single crystals (see Table S1 of the Supplementary Materials part S2). The Li ^+^ ion radius is 0.59 Å, and the Bi^3 +^ ion radius is 0.96 Å. When Li replaces the Bi position in the Bi_2_Se_3_ crystal, the lattice parameters of the *c* axis will decrease. Conversely, the lattice parameters increase for x = 0.02 and 0.08. This result implies that Li may not succeed in replacing Bi and enters the van der Waals layers in between two adjacent quintuple layers (QLs); the larger Li atomic radius (1.54 Å) leads to an increase in the lattice parameters. This result is similar to the *c*-axis lattice parameters of BS magnetism of Upreti et al. in regulating the changes in the *c*-axis lattice parameters of FePS_3_ by Lithium Intercalation [32].

**Figure 2:**
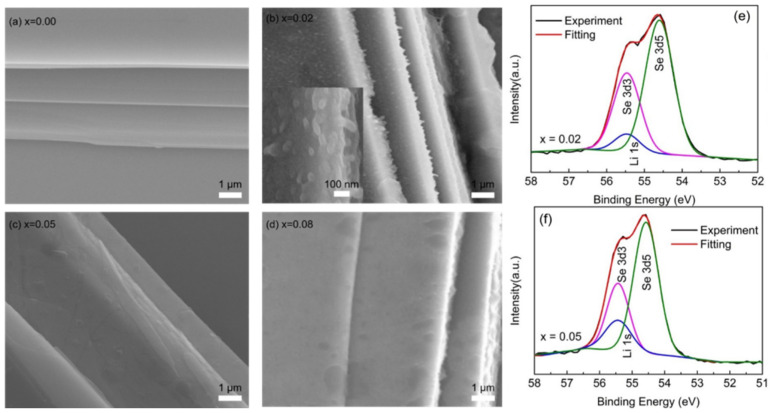
FESEM images of Li_x_Bi_2−x_Se_3_: (**a**) x = 0.00; (**b**) x = 0.02; (c) x = 0.05; (**d**) x = 0.08; (**e**) and (**f**) the XPS spectra of the Li_0.02_Bi_1.98_Se_3_ and Li_0.05_Bi_1.95_Se_3_.

To prove this speculation, the microstructure of the samples was detected by FESEM. The results are shown in Figure 2. Multilayered structures were observed in all samples. Meanwhile, nanoparticles were found to exist in the interlayers of Li-doped samples, as shown in Figure 2b. For the Li (*x* = 0.02 and 0.08) sample, nanoparticles were abundant. The results implied that Li impurities enter the interlayers of samples, and the Li atomic radius is larger than Bi, resulting in an increase in the lattice in the c direction (see the Figure 1b). With the increase in doping amount of 5%, Li impurities diffuse into QLs, the number of Bi atoms that are replaced increases, and the lattice parameters of the sample are reduced. Further, the impurity of Li continues to increase to 8%, more impurity elements may enter the interlayer and inlayer of the crystal, the van der Waals interlayer once again demonstrates nanoclusters, and the lattice parameters increase. The X-ray photoelectron spectroscopy (XPS) of samples further support this inference. From Figure 2e and f, the peak area of Li ^+^ 1*s* (centered on the 54.9 eV) increases with the increase in doping amount, indicating that the relative content of Li ^+^ increases in the samples. This result confirms our prediction based on the XRD data.

**Figure 3:**
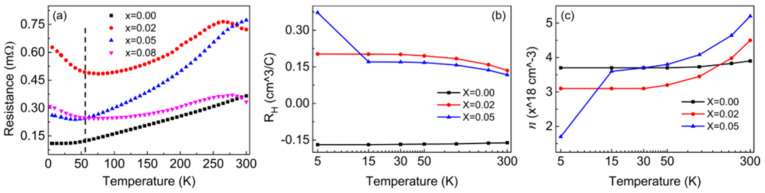
(**a**) Temperature-dependent resistance of Li_x_Bi_2−x_Se_3_ crystals in zero magnetic field (dashed lines are used to indicate the metal-insulation transition temperature of the Li-doped samples, *T* ~55 K); (**b**) the Hall coefficient R_H_ and (**c**) carrier concentration n of Li*_x_*Bi_2−*x*_Se_3_ (*x* = 0, 0.02, 0.05) at different temperatures.

In addition, the carrier type [24], concentration [25], and temperature-dependent characteristics [19,20,21,22] of Bi_2_Se_3_ crystals have a significant influence on the ultrafast dynamics. To analyze the influence of Li impurities on different ultrafast relaxation processes of Bi_2_Se_3_ crystals, the electric transport properties, carrier type, and concentration were measured, as shown in Figure 3. The temperature dependence of the electrical resistance was measured by a PPMS, which shows a typical metal-insulation transition in the low-temperature region for Li-doped samples, as shown in Figure 3a. The metal-insulation transition temperature is marked by the dotted line at approximately 55 K. For *T* ≤ 55 K, the resistance decreases with increasing temperature, showing insulating behavior for the Li_x_Bi_2−x_Se_3_ crystals. For *T* > 55 K, the resistance of the samples increases with temperature and demonstrates a metallic behavior. It is known that the as-grown crystals of Bi_2_Se_3_ display metallic behavior due to the electrons donated by Se vacancies. Chemical doping can drive the Fermi level into the topological gap, leading to nonmetallic behavior [33,34,35]. In Figure 3b, we have obtained Hall coefficients (R_H_) of samples at different temperatures. When Li impurity is introduced in the Bi_2_Se_3_ crystal, R_H_ is changed from negative to positive, indicating that Li impurities provide the holes that compensate for the Se vacancy defects of Bi_2_Se_3_, and the Fermi energy sits within the gap. Insulating behavior is observed at *T* ≤ 55 K in Figure 3a. This phenomenon has been reported in previous studies, which can be explained by the fact that bulk carriers freeze out and surface-state carriers dominate the conductivity in the low-temperature regions, while bulk conductance dominates in the high-temperature regions [36,37,38]. From Figure 3c, the carrier concentration (*n*) of pristine Bi_2_Se_3_ and 2% remained almost constant at *T* ≤ 55 K. When the Li doping amount increases to 5%, *n* increases with temperature at *T* > 50 K. The results indicate that the surface state may be enhanced by Li impurities. According to the previous report by Hong et al., [39] the transport behavior of the Bi_2_Se_3_ is dominated by surface-state electrons at low temperatures, and the bulk carrier activity gradually plays a crucial role as the temperature rises. At *T* ≤ 55 K, the carrier conduction (e.g., phonon) in the bulk carrier is suppressed, and the electron–electron scattering of the materials is dominant in the electric transport behavior [39]. Then, the carriers (e.g., electron) are tuned by Li doping, which leads to the Fermi level adjusting to the gap, and the electrical transport behavior of the material is manifested as an insulating state. For *T* > 55 K, the bulk phonons activity is gradually enhanced, *n* increases rapidly with temperature, the electron–phonon coupling of the bulk carrier dominates the electric transport behavior, and the resistance increases with increasing temperature [39].

**Figure 4:**
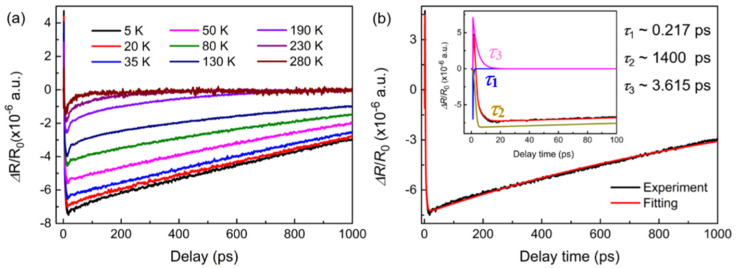
(**a**) Femtosecond (*fs*) transient optical spectroscopy (Δ*R*/*R*(t) signals) measured for Li_0.02_Bi_1.98_Se_3_ at various temperatures ranging from 5 K to 280 K. (**b**) Representative differential reflectivity at 5 K; the red line is a fit to Equation (1). Inset: demonstration of three independent exponential relaxation processes with time constants of *τ*_1_ (blue line), *τ*_2_ (light brown line), and *τ*_3_ (pink line).

The special electric transport mechanism of Li_x_Bi_2−x_Se_3_ is highly related with the dynamic response of nonequilibrium charge carriers in surface states, and it is necessary to further study the ultrafast dynamics of Li_x_Bi_2−x_Se_3_ crystals. At present, fs transient optical spectroscopy has been widely used to study the ultrafast dynamics of topological insulators [40,41,42]. The ultrafast dynamics of Bi_2_Se_3_ has especially been widely studied [14,15,16,17,40,43]. Therefore, femtosecond (*fs*) transient optical spectroscopy was performed to characterize the Li_x_Bi_2−x_Se_3_ crystals; Figure 4a shows the evolution of Li_0.02_Bi_1.98_Se_3_ crystals with increasing temperature, and the *fs* transient optical spectroscopy of other samples (e.g., pure Bi_2_Se_3,_ 5% Li-doped, and 8% Li-doped) are shown in the Supplementary Materials (see the Supplementary Materials part S3). As shown in Figure 4a, the Δ*R*/*R_0_* (*t*) time series was collected from 5 to 280 K, and it is found that the relaxation signal does not return to the equilibrium position at long timescales (1000 ps) below 190 K, which indicates a long relaxation process in the ultrafast response of the samples. And the spectra of all Li-doped samples show similar variation patterns, as shown in Figure S2. However, similar behavior was not observed in Bi_2_Se_3_ crystals [43] (see the Supplementary Materials part S4; the relaxation signals have returned to the equilibrium position at 50 ps), which may be due to Li impurities. Meanwhile, the Δ*R*/*R_0_* (*t*) traces show a rise and multiple decay behavior in the initial few picoseconds (ps), which is typical for Bi_2_Se_3_ crystals [23,43]. The Δ*R*/*R*_0_ (*t*) experimental data for the samples were fitted by three exponential functions to obtain the decay time constants, and Equation (1) can be written as

(1) $$\Delta R/R_{0}\left(t\right)=\sum_{i=1,2,3}A_{i}\exp\left(\frac{t-t_{0}}{\tau_{i}}\right)+C$$

where *A_i_* and *τ_i_* are the amplitude and relaxation time of the decay of hot carriers via different relaxation mechanisms. C is a constant. The three times constants of the three relaxation processes in Li_x_Bi_2−x_Se_3_ crystals can be fitted by Equation (1), where *τ*_1_ is the time constant of electron–electron interaction, *τ*_2_ is the time constant of the carrier recombination process, and *τ*_3_ is the time constant of electron–phonon coupling.

The fitting results for Li_0.02_Bi_1.98_Se_3_ at 5 K are shown in Figure 4b. The decay times *τ*_1_ and *τ*_3_ at 5 K are determined to be approximately 0.217 ps and 3.615 ps, respectively, consistent with the report of Qi et al. [23]. *τ*_2_ is approximately equal to 1400 ps; this result is given only for reference due to experimental test limitations. In fact, the relaxation time of *τ*_2_ could be longer. As shown in Table 1, the three relaxation times of Li_x_Bi_2−x_Se_3_ at different characteristic temperatures are presented.

**Figure 5:**
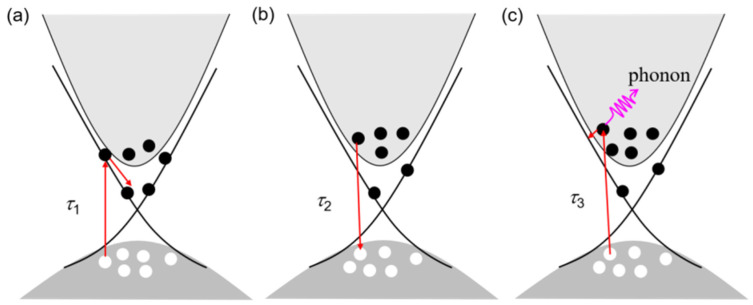
Schematic diagram of three relaxation processes in Li_x_Bi_2−x_Se_3_ crystals: (**a**) *τ*_1_ is the time constant of electron–electron interaction (the electrons in the valence band are excited to the surface state by the laser, and relax to equilibrium along the channel provided by the surface state); (**b**) *τ*_2_ is the time constant of carrier recombination process; and (**c**) *τ*_3_ is the time constant of electron–phonon coupling (the electrons in the valence band are excited to the conduction band by the laser, coupling with the phonon, and relax to the equilibrium).

From Table 1, compared with the pure Bi_2_Se_3_, the ultrafast dynamics of the Li-doped samples is different. For *τ*_1_, especially, it has an advantage in terms of the time scale. And *τ*_2_ represents a new relaxation process. A similar process in Bi_2_Se_3_ thin films has been reported [44,45,46], which was attributed to the carrier recombination process. In this paper, it is speculated that a shallow impurity band exists below the bulk conduction band, which provides a large number of holes to capture hot carriers, the carrier recombination process is slower, and a longer relaxation time is detected. Figure 5b shows a diagram for *τ*_2_. With increasing temperature, the defect carrier motion is intensified, and the hot carrier recombination process is promoted. Therefore, the relaxation time of *τ*_2_ decreases to 190 ~130 ps at 280K. Luo et al. once observed similar Δ*R*/*R*(t) signals in Cu_x_Bi_2_Se_3−y_ samples [47]. Their research results indicated that the negative component in ΔR/R, which is assigned to carrier relaxation in a Dirac cone, is 1.62 ps and 20.5 ps, respectively [47]. The reason for this result might be that different impurity particles are introduced into the Bi_2_Se_3_ system by different methods and concentrations, resulting in different ultrafast carrier dynamics processes and different relaxation times. Thus, Bi_2_Se_3_ materials doped with different elements can be applied in various fields.

**Figure 6:**
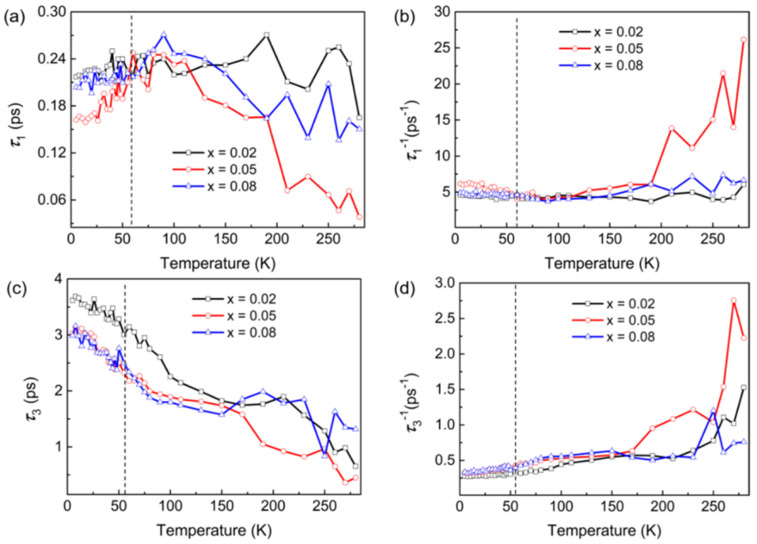
(**a**,**b**) The inverse of the decay time constants (*τ*_1_ and *τ*_1_^−1^) at different temperatures for Li_x_Bi_2−x_Se_3_. (**c**,**d**) The inverse of the decay time constants (*τ*_3_ and *τ*_3_^−1^) at different temperatures for Li_x_Bi_2−x_Se_3_.

According to previous reports, *τ*_1_ and *τ*_3_ can be attributed to the electron–electron interaction and electron–phonon coupling of hot carriers [12,13,19,23,48], which is shown in Figure 5a and Figure 5c, respectively. The fitting data for all samples are shown in Figure 6. Figure 6a and Figure 6b show the temperature dependence of *τ*_1_ for the Li_x_Bi_2−x_Se_3_ crystals, where *τ*_1_ and *τ*_1_^−1^ are the relaxation time and relaxation rate of the electron–electron interaction, respectively. From Figure 6a,b, we find that the value of *τ*_1_ first increases and then decreases with increasing temperature, and the trend for *τ*_1_^−1^ shows the opposite behavior. The temperature dependence of *τ*_1_ and *τ*_1_^−1^ is consistent with the *R*-*T* and carrier concentration. The results show that the electrical transport properties of the Li_x_Bi_2−x_Se_3_ crystals are related to the electron–electron relaxation mechanism. At low temperatures (*T* ≤ 55 K), the electron–electron scattering of the surface state is dominant in the electric transport behavior. However, a large number of impurity carriers are introduced by Li doping, which tune the surface state via hole capture by surface-state electrons. Simultaneously, Li impurities are introduced to regulate the electronic structure and Fermi levels of Bi_2_Se_3_ crystals, leading to the closure of partial carrier channels, an increase in the electron–electron relaxation time and a slowdown in the rate. With increasing temperature, the bulk carriers enhance the electron–electron interaction, and the relaxation becomes faster.

The relaxation process for *τ*_3_ is attributed to electron–phonon coupling, as shown in Figure 6c,d. The relaxation time (*τ*_3_) becomes shorter from 5 K to 280 K. In particular, *τ*_3_^−1^ is very slow at low temperatures. The variation in *τ*_3_ and *τ*_3_^−1^ is also consistent with the *R*-*T* and carrier concentration. The temperature dependence of *τ*_3_ and *τ*_3_^-1^ indicates that the electron–phonon coupling is weak at low temperature (*T* ≤ 55 K).

The result can be explained by the so-called two temperature model (TTM), which is treated as an ultrafast transient of electron–phonon thermalization [18,19,49,50]. The main idea of the TTM is that electron–electron thermalization is much faster than electron–phonon thermalization. However, this basic assumption fails at low temperatures (*T* ≤ 50 K) [50]. Electrons thermalize with the lattice in a characteristic electron–phonon thermalization time, which in metals is typically in the 100 fs–1 ps range [51]. The relationship between the electron–phonon coupling strength and relaxation time can be deduced from the TTM, which is expressed as follows:

(2) $$\lambda \langle \omega^{2}\rangle =\frac{\pi k_{B}T_{e}}{3\hbar \tau_{e-ph}}$$

Here, *λ* is the electron–phonon coupling strength, *τ_e−ph_* is the electron–phonon coupling relaxation time, *w* is the phonon vibration frequency, *k_B_* is the Boltzmann constant, ℏ = *h*/2π, *h* is the Planck constant, and *T_e_* is the electron temperature. When *T* ≤ 55 K, TTM fails, phonons are suppressed by temperature, electron–phonon coupling is weaker, and *τ_e−ph_* is long, as shown in Figure 6c,d. At higher temperature, phonons soften due to lattice expansion, the electron–phonon coupling strength increases, the relaxation time decreases, and the relaxation rate increases. The results further indicate that the electron–electron scattering of the surface state dominates the conductance of the Li_x_Bi_2−x_Se_3_ crystals at low temperature (*T* ≤ 55 K), and the electron–phonon coupling of the bulk carrier dominates the conductance at *T* > 55 K. This result is consistent with the suggestion that surface phonons are less effective in cooling the electron gas in the conduction band [48].

In addition, at *T* ≤ 55 K, no obvious dependence of the three relaxation processes on doping is observed. These results may be related to the small amount of Li replacing Bi. Li ^+^ replaces Bi^3+^, and fewer impurity carriers have less effect on the surface states, which cannot be reflected in the relaxation process dominated by the surface state at low temperature. However, the 5% Li-doped samples still showed differences, which further indicated that Li replacing Bi was more favorable for surface-state enhancement. At a higher temperature (*T* >150 K), the relaxation rate of Li_0.05_Bi_1.95_Se_3_ becomes obviously faster. This result is consistent with the carrier concentration at the different temperature, which suggested the response of *τ*_1_ and *τ*_3_ may be related to the carrier concentration. Previously, several groups have reported that carrier dynamics of TI can be influenced by carrier concentration [44,46,52,53]. These results indicate that Bi is replaced, which shows an advantage in the ultrafast relaxation process that is dominated by the bulk carrier. Furthermore, it has also been reported that electron–electron scattering and Mott modes coexist at low temperatures. When the Mott range-varying transition mode is dominant, electrons can jump between energy levels that are close in energy but far apart, thereby causing the metal-insulator transition [36,54]. At this point, we speculate that the minor changes in *τ*_1_ and *τ*_3_ around 55K may also be related to the metal-insulation transition of the material.

## 4. Conclusions

In this paper, Li_x_Bi_2−x_Se_3_ crystals were synthesized by the self-flux method. The crystalline structure was characterized by XRD and SEM, and the results showed that Li impurities are introduced into Bi_2_Se_3_ crystals. The *R*-*T* curve and the data of Hall measurements show that the carriers in Bi_2_Se_3_ can be tuned by the Li impurities. Femtosecond (*fs*) transient optical spectroscopy (Δ*R*/*R*(t) signals) was measured and analyzed to obtain the ultrafast dynamics parameters. The temperature dependence for the relaxation rates of the electron–electron interaction and electron–phonon coupling is consistent with the results of electrical transport. The results indicate that electron–electron interaction is dominated by free carriers at low temperatures (*T* ≤ 55 K), and electron–phonon coupling is dominated by bulk carriers at *T* > 55 K.

## Figures and Tables

**Table 1:** The three relaxation times of the Li_x_Bi_2−x_Se_3_ at different characteristic temperatures.

Sample	5 K	35 K	55 K	150 K	280 K
Bi_2_Se_3_	***τ***_1_~1.61 ps	***τ***_1_~1.097 ps	***τ***_1_~1.067 ps	***τ***_1_~1.034 ps	***τ***_1_~0.915 ps
	***τ***_2_~/	***τ***_2_~/	***τ***_2_~/	***τ***_2_~/	***τ***_2_~/
	***τ***_3_~2.44 ps	***τ***_3_~1.520 ps	***τ***_3_~1. 514 ps	***τ***_3_~1.016 ps	***τ***_3_~1.005 ps
Li_0.02_Bi_1.98_Se_3_	***τ***_1_~0.217 ps	***τ***_1_~0.230 ps	***τ***_1_~0.224 ps	***τ***_1_~0.232 ps	***τ***_1_~0.165 ps
	***τ***_2_~1400 ps	***τ***_2_~1540 ps	***τ***_2_~1124 ps	***τ***_2_~800 ps	***τ***_2_~180 ps
	***τ***_3_~3.615 ps	***τ***_3_~3.474 ps	***τ***_3_~3.004 ps	***τ***_3_~1.822 ps	***τ***_3_~0.654 ps
Li_0.05_Bi_1.95_Se_3_	***τ***_1_~0.162 ps	***τ***_1_~0.176 ps	***τ***_1_~0.210 ps	***τ***_1_~0.181 ps	***τ***_1_~0.038 ps
	***τ***_2_~1400 ps	***τ***_2_~1380 ps	***τ***_2_~1500 ps	***τ***_2_~600 ps	***τ***_2_~130 ps
	***τ***_3_~3.032 ps	***τ***_3_~2.731 ps	***τ***_3_~2.324 ps	***τ***_3_~1.740 ps	***τ***_3_~0.449 ps
Li_0.08_Bi_1.92_Se_3_	***τ***_1_~0.204 ps	***τ***_1_~0.208 ps	***τ***_1_~0.221 ps	***τ***_1_~0.221 ps	***τ***_1_~0.156 ps
	***τ***_2_~1200 ps	***τ***_2_~1660 ps	***τ***_2_~1860 ps	***τ***_2_~850 ps	***τ***_2_~186 ps
	***τ***_3_~2.986 ps	***τ***_3_~2.670 ps	***τ***_3_~2.495 ps	***τ***_3_~1.576 ps	***τ***_3_~1.315 ps

## Data Availability

The original contributions presented in this study are included in the article; further inquiries can be directed to the corresponding author.

## Supplementary Materials

The following supporting information can be downloaded at: https://www.mdpi.com/article/10.3390/nano15131010/s1, Figure S1: The samples of Li*_x_*Bi_2−*x*_Se_3_ crystals; Figure S2: (a) and (b) Representative Δ*R*/*R*_0_(*t*) time series of single crystal Li*_x_*Bi_2−*x*_Se_3_ at 5–280 K. (c) and (d) Representative Δ*R*/*R*_0_(*t*) time series of single crystal Li*_x_*Bi_2−*x*_Se_3_ within 15 ps; Figure S3: (**a**) Representative Δ*R*/*R*_0_(*t*) time series of single crystal Bi_2_Se_3_ at 5–280 K. (b) Δ*R*/*R*_0_(*t*) time series at 5 K and the fitting with Equation (1); Table S1: Li content for all samples [33,55].
