# Supplementary material for: Doping Tuned the Carrier Dynamics in Li-Doped Bi2Se3 Crystals Revealed by Femtosecond Transient Optical Spectroscopy
Source: Nanomaterials (Basel). 2025 Jun 30;15(13):1010. doi: 10.3390/nano15131010 (PMC12250898; doi:10.3390/nano15131010)
Supplement: Supplementary file 1 [file nanomaterials-15-01010-s001.zip › nanomaterials-3702458-supplementary.pdf]

nanomaterials

Supplementary Materials

Doping Tuned the Carrier Dynamics in Li-Doped Bi_2Se_3 Crystals Revealed by Femtosecond Transient Optical Spectroscopy

Qiya Liu [1,2], Min Zhang[3,*], Xinsheng Yang[2,*], Tixian Zeng [1,4] and Minghu Pan [5]

[1] College of Optoelectronic Technology, Chengdu University of Information Technology, Chengdu 610225, China liuqiya@cuit.edu.cn (Q.L.); zengtx@cuit.edu.cn (T.Z.).
[2] Superconductivity and New Energy R&D Center, School of Physical Science and Technology, Southwest Jiaotong University, Chengdu 610031, China xsyang@swjtu.edu.cn (X.Y.).
[3] School of Physics and Astronomy, China West Normal University, Nan Chong 637002, China
[4] Dazhou Industrial Technology Research Institute, Dazhou, 635000, China
[5] School of Physics and Information, Shanxi Normal University, Xi'an 710119, China, minghupan@snnu.edu.cn (M.P.).
* Correspondence: zmzmi1987@cwnu.edu.cn (M.Z.), xsyang@swjtu.edu.cn (X.Y.)

S1. Test Samples Information

In this paper, the samples were prepared by self-flur method, as shown in Figure S 1a. The test samples are exfoliating flakes from the $Li_xBi_{2-x}Se_3$ block in Figure S1 a, and the size is 3 mm*1.5 mm, the thickness is about 800 nm, shown in Figure S1b.

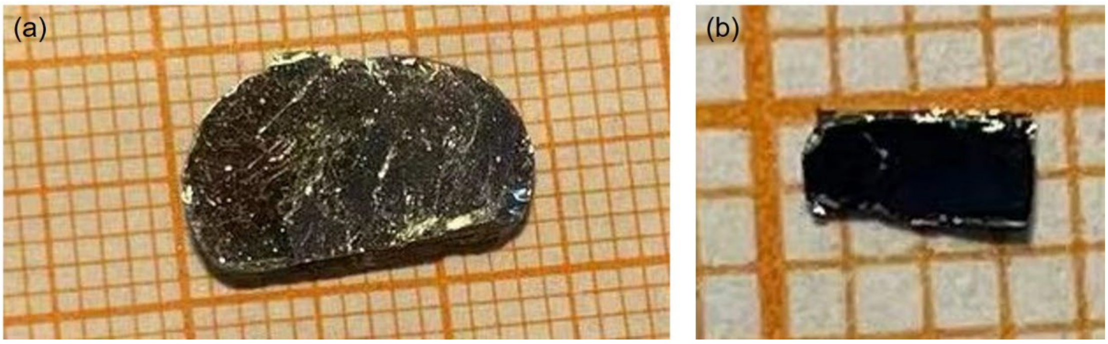

Figure S1 The samples of $Li_xBi_{2-x}Se_3$ crystals.

S2. Composition Analysis of Test Samples

In addition, the Li atoms are light and wt% is small in the samples, the EDX cannot be used to detect relevant information. The results of ICP-OES indicated that the content of Li was consistent with the stoichiometric ratio in the $Li_xBi_{2-x}Se_3$ single crystals, shown in Table S1. And other significant impurities were not detected in the samples. It is suggested that these nanoclusters are aggregated caused by Li atom.

Table S1. Li content for all samples.

**Content**	Nominal (wt %)			ICP-OES (bulk) Li
**x**	Bi	Se	Li	(wt %)
0.02	63.565	36.412	0.023	0.020
0.05	63.207	36.742	0.051	0.048
0.12	62.307	37.561	0.132	0.130

S3. The $\Delta R/R_0(t)$ Time Series of $Li_xBi_{2-x}Se_3$ Single Crystal

In this manuscript, we also tested the *fs* transient optical spectroscopy of other samples, as shown in Figure S2a and b, the $\Delta R/R_0\,(t)$ time series of 5% and 8% was collected from 5–280 K. Simultaneously, the initial signals of $\Delta R/R_0(t)$ were shown in Figure S2c and (d), and an *fs* laser pump pulse induces a fast rise in $\Delta R/R_0$ within ~300 fs, followed by a combination of non-oscillation and oscillation decay processes. The test result is similar to the pure Bi_2Se_3 crystals.

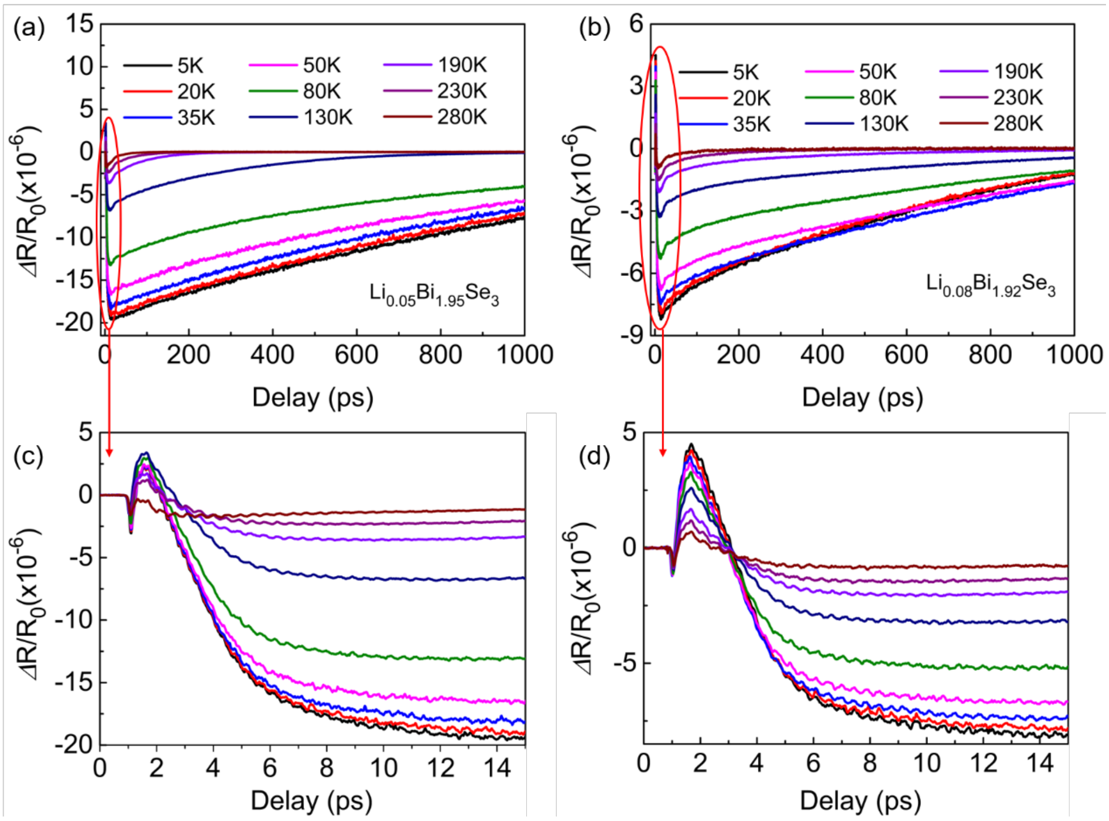

Figure S2 (a) and (b) Representative $\Delta R/R_0(t)$ time series of single crystal $Li_xBi_{2-x}Se_3$ at 5–280 K. (c) and (d) Representative $\Delta R/R_0(t)$ time series of single crystal $Li_xBi_{2-x}Se_3$ within 15 ps.

S4. The $\Delta R/R_0(t)$ Time Series of Single Crystal Bi_2Se_3

In the previous report, we have studied the transient reflectivity of Bi_2Se_3 crystals, and the test results are shown in Figure S3. And the results have been published in Appl. Phys. lett. 115 (2019). We collect $\Delta R/R_0$ time series from 5--280 K, and Figure S4a shows representative traces. An *fs* laser pump pulse induces a fast rise in $\Delta R/R_0$ within ~300 fs, followed by a combination of non-oscillation and oscillation decay processes. The initial fast rise is due to the excitation of hot carriers,[1] and the oscillation decay component is attributed to excited coherent phonons in Bi_2Se_3. The decaying ultrafast oscillation is superimposed on a nonoscillating background decay.[1,2] As temperature rises, the ultrafast oscillation signal weakens, and the relaxation time shortens.

Time series $\Delta R/R_0\,(t)$ can be described with two decay processes,

$$\Delta R / R_0 = \sum_{i=1,2} A_i \exp\left(-\frac{t-t_0}{\tau_{Ai}}\right) + \sum_{i=1,2} B_i \exp\left(-\frac{t-t_0}{\tau_{Bi}}\right) \sin\left(\omega_i t + \phi_i\right) + C \qquad \text{(S1)}$$

Here τ is time. A_i and τ_{Ai} are amplitude and relaxation time of the ith non-oscillation component of the decay of hot carriers. B_i and τ_{Bi}, ω_i and ϕ_i are amplitude, dephasing time, angular frequency, and initial phase of the ith oscillation component of the decay of coherent phonons. C is a constant.

As shown in Figure S3b, the decay times τ_1 and τ_2 at 5 K are determined to be approximately 1.61 ps and 2.44 ps, respectively.

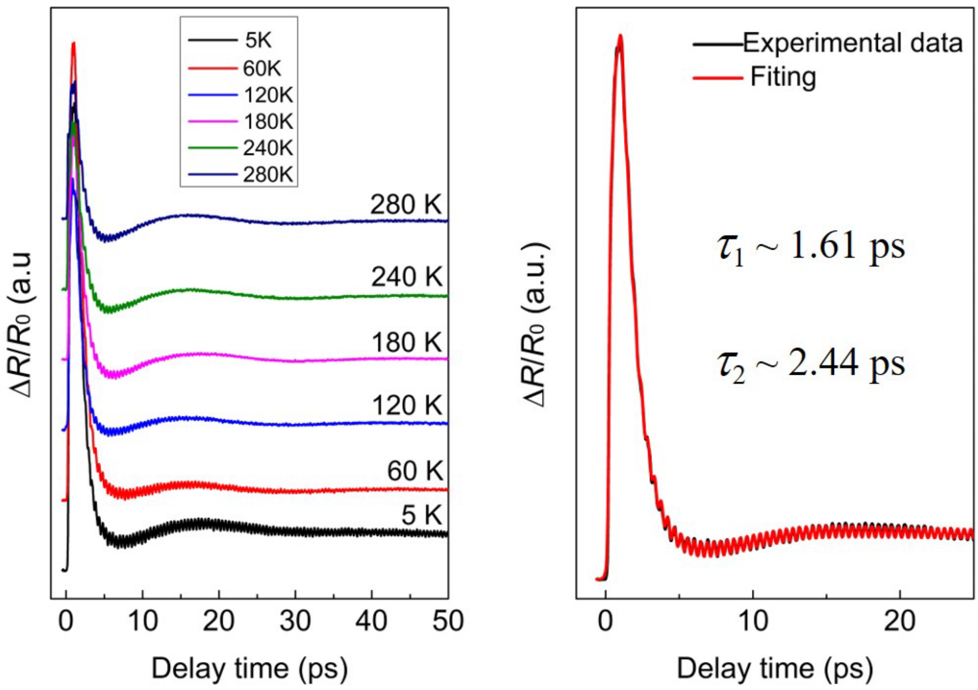

Figure S3 (**a**) Representative $\Delta R/R_0(t)$ time series of single crystal Bi_2Se_3 at 5–280 K. (b) $\Delta R/R_0(t)$ time series at 5 K and the fitting with Equation (1).

Reference

1. J. G. Checkelsky, Y. S. Hor, R. J. Cava, et al. Bulk Band Gap and Surface State Conduction Observed in Voltage-Tuned Crystals of the Topological Insulator Bi_2Se_3. *Physical Review Letters*, **2011**, 106:196801.
2. J. L. Wang, L. Guo, C. Ling, et al. Carrier dynamics in femtosecond-laser-excited bismuth telluride. *Physical Review* B, **2016**, 93:155306.
